# *CYP1A1 *gene polymorphisms increase lung cancer risk in a high-incidence region of Spain: a case control study

**DOI:** 10.1186/1471-2407-10-463

**Published:** 2010-08-30

**Authors:** Carmen San Jose, Agustin Cabanillas, Julio Benitez, Juan Antonio Carrillo, Mercedes Jimenez, Guillermo Gervasini

**Affiliations:** 1 Merida Hospital, Research Unit, 06008 Merida, Spain; 2 Department of Pharmacology, Medical School, University of Extremadura, Av. Elvas s/n, 06071 Badajoz, Spain

## Abstract

**Background:**

A rural region in south-west Spain has one of the highest lung cancer incidence rates of the country, as revealed by a previous epidemiological 10-year follow-up study. The present work was undertaken to ascertain the role of *CYP1A1 *gene polymorphisms and their interaction with tobacco smoking in the development of the disease in this location.

**Methods:**

One-hundred-and-three cases of lung cancer and 265 controls participated in the study. The participants were screened for the presence of four *CYP1A1 *polymorphisms, namely *MspI*, Ile462Val, T3205C, and Thr461Asn. Lung cancer risk was estimated as odds ratios (OR) and 95% confidence intervals (CI) using unconditional logistic regression models adjusting for age, sex, and smoking.

**Results:**

The distribution of the variant *CYP1A1 *alleles was different from that described for other Caucasian populations, with CYP1A1*2A showing an uncommonly high frequency (p < 0.01). The CYP1A1*2B allele (carrying *MspI *and Ile462Val mutations) was strongly associated with high lung cancer risk (OR = 4.59, CI:1.4-12.6, *p *<0.01). The Ile462Val polymorphism was also shown to increase the risk for the disease (OR = 4.51, CI:1.8-11.9; *p *<0.01) and particularly for squamous-cell (OR = 5.01; CI: 1.6-14.3, p < 0.01) and small-cell lung carcinoma (SCLC) (OR = 6.97, CI: 1.2-81.3; *p *= 0.04). Moreover, the Thr461Asn polymorphism was found to be associated with SCLC in a Caucasian population for the first time to our knowledge (OR = 8.33, CI: 1.3-15.2, *p *= 0.04).

**Conclusion:**

The results suggest that *CYP1A1 *polymorphisms contribute to increase lung cancer susceptibility in an area with an uncommon high incidence rate.

## Background

In the late eighties, a group of oncologists working at the Merida hospital, which is located in an agricultural region situated in south-west Spain, started to notice the unusually high number of lung cancer patients being diagnosed. This prompted the creation of a local lung cancer registry in accordance with the guidelines issued by the International Agency for Research on Cancer (IARC) and the International Association of Cancer Registries (IACR). Quite shockingly, in a 10-year follow-up this registry revealed one of the highest standardized incidence rates of male lung cancer in Spain (58 cases per 100 000 inhabitants in the 1986-1990 period, Cabanillas et al., unpublished observations). This figure, which has remained high over the years that followed [1], is much higher than the Spanish mean (43 cases/100 000 inhabitants in the same period) and similar to that of heavily industrialized and mining areas of northern Spain.

Tobacco smoking has long been established as a risk factor for lung cancer, even though fewer than 20% of smokers develop the disease. Tobacco smoke contains several carcinogens including polycyclic aromatic hydrocarbons (PAHs), N-nitrosamines, and heterocyclic amines [2], which undergo biotransformation via a number of metabolic routes. Cytochrome P450 (CYP) isozymes activate these environmental pollutants to yield highly reactive substances that bind to DNA, forming adducts involved in the initiation of carcinogenesis [3,4]. Within the CYP system, CYP1A1 plays a major role as a carcinogen activating enzyme. Unlike most CYP enzymes, CYP1A1 is mainly expressed in extrahepatic tissues, including the lung, where it metabolizes and is markedly induced by PAHs [5,6]. Elevated CYP1A1 inducibility is associated with pulmonary PAH-related DNA adduction [7] and high lung cancer risk [8,9]. Both the formation of these PAH-DNA adducts and CYP1A1 expression in human lung tissue are highly variable [10-13], possibly due to differing exposure to environmental factors and to genetic polymorphisms affecting the CYP1A1 gene locus.

A number of *CYP1A1 *allele variants have been associated with a higher inducibility and/or activity of the enzyme, and hence higher pulmonary PAH-related DNA adduction [7]. The first variant allele identified was CYP1A1*2A (also known as *MspI *or *m1 *polymorphism) and is found in 5% of Caucasians [14]. CYP1A1*2C (Ile462Val or *m2 *polymorphism) is rare in Caucasians and is mostly detected in linkage disequilibrium with CYP1A1*2A [15]. The combination of both variants is referred to as CYP1A1*2B (for a complete list of *CYP1A1 *variant alleles see http://www.cypalleles.ki.se/cyp1a1.htm). CYP1A1*3, consisting of a T3205C base change (*m3*), also seems to confer enhanced enzyme activity, although it is extremely uncommon in Caucasians [14]. Finally, CYP1A1*4, a Thr461Asn (*m4*)amino acid change that is detected in Caucasians with a frequency of roughly 3%, has also been related to greater enzyme catalytic efficiency [16].

These *CYP1A1 *polymorphisms have been extensively studied with regard to lung cancer risk. However, whereas some studies report increased risk in the presence of some of the mutations [17-21], there are many other contradictory results and ethnic differences [22-25], which has led to the perception that the findings have been inconsistent [22].

The goal of the present study was therefore to determine whether *CYP1A1 *polymorphisms and their interaction with smoking may play a role in the aforementioned extraordinarily elevated lung cancer incidence in our population.

## Methods

### Study design

We conducted a case-control study in the health district of Merida (Spain), which gives health coverage to a population of 156 000, in order to establish whether *CYP1A1 *polymorphisms may contribute to the elevated prevalence of lung cancer in the region. Cases were Caucasian patients first diagnosed with lung cancer at the Merida hospital. This hospital is the only referral cancer health centre for the region, and thus all cancer disease diagnoses and patient follow-up are carried out in this hospital. The diagnosis was based on histological analyses of endoscopic biopsies and/or surgical resection specimens. The patients were selected by non-probability consecutive sampling with no restrictions for age, sex, or tumour grade (Eastern Cooperative Oncology Group, ECOG). Information on tumour extension, grade and histological type was extracted from clinical records and the files of the Merida Hospital Pathological Anatomy Service. Controls were cancer-free individuals admitted to the Hospital Trauma Service and matched to the cases by sex and age (± 5 years). All the participants were interviewed by trained Hospital personnel to collect data on anthropometric characteristics, family history and details of smoking habits.

Each subject was aware of the purpose of the study, and gave oral and written informed consent for participation. The study was approved by the Ethics Committee of the University of Extremadura (Badajoz, Spain) and was conducted in accordance with the Declaration of Helsinki and its subsequent revisions

### Genotyping

Blood samples were drawn from all participants and immediately stored at -80°C until genotype analysis. Genomic DNA was isolated from peripheral blood leukocytes in 2-ml aliquots of the whole-blood sample with a Qiagen blood midi kit (Qiagen Inc., Chatsworth, CA).

The *MspI *polymorphism was detected by PCR amplification followed by digestion with *MspI *restriction enzyme as described by Cascorbi et al [26]. In order to identify the Ile462Val and Thr461Asn mutations, a single PCR amplicon was digested with *BsrDI *or *BsaI *restriction enzymes, respectively [26]. Finally, the T3205C polymorphism was detected following the method reported by Hayashi and co-workers [27].

Previously sequenced samples were used as negative and positive controls to rule out possible genotyping errors. Likewise, the analysis all mutant homozygous samples and 20% of heterozygotes was duplicated and confirmed by direct sequencing with 100% concordance (ABI3700 DNA Analyzer; Perkin-Elmer/Applied Biosystems).

### Statistical analyses

Power analysis retrieved a sample size of 94 cases and 94 patients necessary to detect an OR of 4 with a double-tale significant level of α = 0.05 and χ^2 ^= 0.80, assuming a global prevalence of heterozygotes for the CYP1A1*2B and CYP1A1*2A alleles of 6.4% and 10.5% respectively in Caucasian population [14].

Hardy-Weinberg equilibrium was tested for the different *CYP1A1 *genotype frequencies in controls using Pearson's chi-squared test with one degree of freedom. In order to estimate the association between *CYP1A1 *variant alleles and lung cancer risk, a stepwise conditional multiple logistic regression analysis was performed with enrolled threshold α ≤ 0.20 and excluded threshold α ≥ 0.05. The odds ratios (OR) with 95% confidence intervals (CI) were thus calculated for each significant risk factor. The models were adjusted for age, sex, and cumulative tobacco consumption unless stated otherwise. To avoid collinearity in the regression analysis of the risk, and to enhance the statistical power of the study, participants were divided into 4 genotype groups according to the mutations carried. Group 1 consisted of the homozygous wild-type subjects (CYP1A1*1/*1 genotype), Group 2 of subjects with the *MspI *polymorphism (CYP1A1*1/*2A genotype), Group 3 of subjects with the Ile462Val mutation (CYP1A1*1/*2B, CYP1A1*3/*2B, and CYP1A1*1/*2C genotypes), and Group 4 of subjects with the Thr461Asn polymorphism (CYP1A1*1/*4, CYP1A1*4/*4, and CYP1A1*2A/*4 genotypes). The CYP1A1*1/*3 genotype was present in only one case and two controls, and hence was eliminated from the statistical analysis.

Cumulative tobacco consumption was quantified in pack-years (defined as the number of packs of 20 cigarettes smoked per day multiplied by the number of years of smoking). The median of pack-years (50) was used as cut-point for the stratification analysis. Subjects with a pack-years value above 50 were considered heavy smokers, subjects with values less than or equal to 50 moderate smokers, and subjects with values less than 0.2 pack-years or who had never smoked were considered non-smokers. Finally, subjects who had quit smoking more than a year before the diagnosis (cases) or intervention (controls) were regarded as previous smokers.

Quantitative variables such as age and cumulative tobacco consumption (pack-years) were compared by Student's t-test. The chi-squared or Fisher's exact tests were used to compare categorical variables between cases and controls (sex, smoking status, family history of tumours, and allele and genotype frequencies).

Statistical analyses were conducted using the SPSS software package version 9.0 for Windows (SPSS Chicago, IL) and Statistical Analysis System version 9.1.3 (SAS Institute). In all instances, results were considered significant at the two-sided *p *< 0.05 level.

## Results

In the period of study, 427 subjects (115 cases and 312 controls) fulfilled the inclusion criteria. Fifty-nine of them rejected participating in the study, therefore a total of 368 individuals (103 cases and 265 controls) were finally included. Mean age was 2.1 years lower in control subjects (66.0 ± 10.7 *vs *63.9 ± 12.9; *p *= 0.019), whereas the percentage of men was similar between both study groups (95.1% *vs *95.9%; *p *= 1).

Table 1 shows that the proportion of current and heavy smokers and the number of pack-years was significantly higher among cases (*p *< 0.001). In addition, patients who were former smokers had quit smoking more recently than controls (11.3 ± 9.6 *vs *17.2 ± 12.1 years, *p *= 0.03). Lastly, a history of first-degree family cancer was also reported more frequently among cases (*p *< 0.001, Table 1).

**Table 1:** Characteristics of the 103 lung cancer patients and 265 controls.

	Cases, N (%)	Controls, N	*p *value
Smoking status			< 0.001 ^ξ^
Never	6 (5.8)	69 (26.1)	
Former	36 (34.9)	128 (48.2)	
Current	61 (59.2)	68 (25.7)	
Pack-years (PY)	61.2 ± 28.5	29.9 ± 21.4	< 0.001 ^‡^
Current and Former Smoking			< 0.001 ^ξ^
Moderate (≤50 PY)	30 (29.1)	137 (51.8)	
Heavy (>50 PY)	67 (65)	59 (22.1)	
Family history of tumours			< 0.001 ^ξ^
Yes	47 (45.6)	66 (24.9)	
No	56 (54.4)	199 (75.1)	
Histological tumour type			
Squamous cell carcinoma	49 (47.6)		
Large cell carcinoma	23 (22.3)		
Adenocarcinoma	16 (15.5)		
Small cell carcinoma	15 (14.6)		

* Plus-minus values are means ± standard deviation. Because of rounding, percentages may not total 100.

^‡ ^Student's t test.

^ξ ^Pearson's χ^2 ^analysis.

^γ^First-degree family.

With regard to the genotype analysis, the frequencies of the 4 mutations in the control group (9.9%, 1.1%, 0.5%, and 3.9% for *MspI*, Ile462Val, T3205C, and Thr461Asn, respectively) were similar to those previously described in Caucasians [26]. However, the distribution of the variant alleles was different. In the control group, CYP1A1*2A had a frequency that was uncommonly high for Caucasians (8.5%, *p *< 0.01 *vs *other Caucasian populations [14,26]). In contrast, CYP1A1*2B and CYP1A1*2C alleles were under-represented although the difference did not reach statistical significance.

The analysis of differences between cases and controls showed the CYP1A1*2B allele to be associated with increased lung cancer risk (OR = 4.59; CI: 1.4-12.6, *p *< 0.01, Table 2).

**Table 2:** Distribution of *CYP1A1 *alleles in cases and controls.

Allele	Controls, N (%)	Cases, N (%)	OR (CI)	*p *value
CYP1A1*1	452 (85.3)	168 (81.6)	1.0 (Ref.)	
CYP1A1*2A	45 (8.5)	11 (5.3)	0.62 (0.3-1.2)	0.20
CYP1A1*2B	7 (1.3)	12 (5.8)	4.59 (1.4-12.6)	< 0.01
CYP1A1*2C	1 (0.2)	1 (0.5)	Nc	
CYP1A1*3	2 (0.4)	2 (1)	Nc	
CYP1A1*4	23 (4.3)	12 (5.8)	1.32 (0.7-2.9)	0.29

N: Number of alleles

OR (CI): Odds ratio with 95% confidence interval

nc: Non-calculable

Nine different *CYP1A1 *genotypes were detected in the study population (Table 3). Their frequencies were consistent with Hardy-Weinberg equilibrium in the control group (χ^2^= 0.58, *p *= 0.91). A total of 189 (71.3%) controls and 68 (66.0%) cases were carriers of the homozygous wild-type genotype. The two commonest variant genotypes were CYP1A1*1/*2A and CYP1A1 *1/*2B, the latter being four times more frequent in cases (10.7%) than in controls (2.6%, *p *< 0.01, Table 3). There were no subjects who were homozygous for the CYP1A1*2A, *2B, or *3 alleles, and only 1 case and 1 control carried the CYP1A1*4/*4 genotype.

**Table 3:** *CYP1A1 *genotype frequencies observed.

	Controls		Cases		
*CYP1A1 *genotype	N (%)	CI	N (%)	CI	*^ξ ^p*
*1/*1	189 (71.3)	65.5-76.7	68 (66.0)	55.9-75.1	0.371
*1/*2A	44 (16.6)	12.3-21.7	10 (9.7)	2.6-8.7	0.102
*1/*2B	7 (2.6)	1.1-5.4	11 (10.7)	2.7-9.4	< 0.01
*1/*2C	1 (0.4)	0-2.1	1 (1.0)	0-5.3	nc
*1/*3	2 (0.7)	0.1-2.7	1 (1.0)	0-5.3	nc
*1/*4	20 (7.6)	-	9 (8.7)	4.1-15.9	0.829
*2A/*4	1 (0.4)	0-2.1	1 (1.0)	0-5.3	nc
*2B/*3	0 (0)	-	1 (1.0)	0.5.3	nc
*4/*4	1 (0.4)	0-2.1	1 (1.0)	0.5.3	nc
Total	265		103		

Because of rounding, percentages may not total 100.

N: Number of subjects

nc: non-calculable

^ξ ^Pearson's χ^2 ^analysis

After adjusting for age, sex, and smoking, those subjects harbouring the Ile462Val mutation (*2B/*1, *2B/*3 and *2C/*1 genotypes) were at higher lung cancer risk, whether considering all cases combined (OR = 4.51, CI: 1.8-11.9; *p *< 0.01), or the small-cell lung carcinoma (SCLC) (OR = 6.97, CI: 1.2-81.3; *p *= 0.04), and squamous-cell carcinoma subgroups (OR = 5.01, CI: 1.6-14.3; *p *< 0.01) (Table 4). Finally, subjects carrying the Thr461Asn polymorphism (CYP1A1*4 allele) were also associated with a higher risk of SCLC (OR = 8.33, CI: 1.1-15.2, *p *= 0.04) (Table 4).

**Table 4:** Odds ratios (OR)* and 95% confidence intervals (CI) for lung cancer risk according to *CYP1A1 *genotype groups and stratification by histological types.

	*1/*1	*1/*2^a^	*1/2B *2B/*3 *1/*2C	*1/*4 *4/*4 *2A/*4
Controls, N (%)	189 (71.9)	44 (16.7)	8 (3.0)	22 (8.4)
All cases, N (%)	68 (66.7)	10 (9.8)	13 (12.7)	11 (10.8)
OR (CI)	1.0	0.51 (0.3-1.2)	4.51 (1.8-11.9)	1.29 (0.6-2.9)
*p*		0.18	< 0.01	0.29
				
Squamous-cell, N (%)	35 (71.4)	5 (10.2)	7 (14.3)	2 (4.1)
OR (CI)	1.0	0.51 (0.2-1.6)	5.01 (1.6-14.3)	0.49 (0.1-2.4)
*p*		0.37	< 0.01	0.40
				
Adenocarcinoma, N (%)	10 (66.7)	1 (6.7)	2 (13.3)	2 (13.3)
OR (CI)	1.0	0.41 (0.04-2.91))	4.71 (0.8-27.3)	1.62 (0.3-7.7)
*p*		0.51	0.10	0.65
				
SCLC, N (%)	9 (60)	-	2 (13.3)	4 (26.7)
OR (CI)	1.0	nc	6.97 (1.2-81.3)	8.33 (1.1-15.2)
*p*		nc	0.04	0.04
				
LCLC, N (%)	14 (60.9)	4 (17.4)	2 (8.7)	3 (13)
OR (CI)	1.0	1.01 (0.3-3.1)	2.7 (0.5-14.6)	1.68 (0.4-5.2)
*p*		1	0.24	0.75

*ORs were adjusted for age, sex, and smoking (non/moderate/heavy), taking the CYP1A1*1/*1 genotype as reference. Genotype groups were generated as described in the *Methods *section.

SCLC: Small-cell lung carcinoma.

LCLC: Large-cell lung carcinoma.

nc: non-calculable.

After stratification of the population according to cumulative tobacco dose (non-smokers, moderate smokers, and heavy smokers), none of the 4 different genotype groups analyzed was significantly associated to higher lung cancer risk. In moderate smokers the observed OR (CI) were 0.61 (0.03-2.0), 3.98 (0.3-43.8) and 1.11 (0.1-8.0) for subjects carrying the *MspI*, Ile462Val and Thr461Asn polymorphisms, respectively. With regard to heavy smokers, OR (CI) values for the same genotype groups were 1.08 (0.1-7.5), 3.12 (0.4-24.9) and 2.5 (0.2-19.7), respectively. The low number of non-smokers did not allow performing risk analyses for any of the genotype groups.

## Discussion

This study was designed to determine whether genetic polymorphisms in the *CYP1A1 *gene, an activator of carcinogens present in tobacco smoke, could play a role in the extremely high lung cancer incidence rate observed in a rural region of Southern Spain.

Results show that the pattern of combinations of mutations yielding the different *CYP1A1 *variant alleles in the present study differs from that reported for Caucasians. Several Spanish populations have been shown in the past particularities regarding the presence of polymorphisms in other CYP enzymes [28,29]. The reason seems to be the occurrence of gene flow from North Africa across the Strait of Gibraltar that has caused greater intermingling of gene pools than in most European-derived populations [30,31]. Thus, the CYP1A1*2A mutant allele frequency in our population was significantly greater than that observed in 2 previous large-scale studies with Caucasians [14,26]. It is noteworthy that CYP1A1*2A is far commoner in Africans, in whom its frequency is over 20% [14], so that the aforementioned gene flow could be behind the high frequency observed in our population. Furthermore, the CYP1A1*2B and CYP1A1*2C frequencies in the controls were lower than those reported for Caucasians (although the differences did not reach statistical significance), but mirrored those observed in Africans [14].

We then tried to determine whether these particularities regarding the presence of *CYP1A1 *variants were translated into different susceptibilities to lung cancer in our population. Most notably, subjects carrying the 462Val variant accounted for 12.7% of cases but only 3% of controls. This polymorphism has consistently been associated with lung cancer risk in Asian subjects [32,33], while results in Caucasians have been more variable [19-21,23,25,34]. The reason for the controversy in the literature seems to be smoking, since the mutation is believed to be important among light- and non-smokers but not among heavy-smokers [19,20,23,35].

A limitation of the present study was the low number of nonsmokers found among patients, which, for instance, precluded a full assessment of the aforementioned interaction between smoking and the Ile462Val polymorphism. In addition, smoking is commonplace in our rural population, which made the median of pack-years used to stratify the subjects into moderate and heavy smokers extremely high (50). In comparison, the median in a similar Swedish study was 21 [25]. Thus, the vast majority of participants in the study were smokers who had been exposed to high amounts of tobacco smoke throughout their lifetimes. The lack of patients with low exposure to tobacco smoke in our region was probably also the reason why none of the 4 genotype groups assessed showed significant ORs after stratification by cumulative tobacco dose.

In the present study, there was a particularly marked impact of the 462Val mutation on the susceptibility to SCLC, showing a significant OR of 6.97. This is coherent with another case-control study that analyzed the association of the Ile462Val polymorphism with several lung cancer histological types, and found a slightly higher OR (9.35) for SCLC [35]. Other reports, however, indicate that the risk is higher for squamous-cell carcinoma [19] (an association that was also observed in our study) or non-small cell carcinomas [36]. It is of note, however, that none of the two latter studies included SCLC cases among the participants.

Another important finding of the present study was the positive association of the Thr461Asn polymorphism (CYP1A1*4 allele) with SCLC risk, since this variant was 3 times more frequent among these patients than in the control group (OR = 8.33). This is the first time to our knowledge that this allele has been related to high lung cancer risk in a Caucasian population, since similar results have only been observed in Indians [37], African-Americans [38] or Hispanics [39]. In Caucasians, Vineis et al. recently detected a positive allele-disease association, but only where the variant occurred in combination with a number of other *CYP1A1 *and *GST *polymorphisms [21].

Unlike our findings for the Ile462Val and Thr461Asn polymorphisms, the results showed no association of the *MspI *polymorphism (CYP1A1*2A allele) with lung cancer, which is consistent with earlier reports in Caucasians [40-42]. Recent studies have found that an increased risk may occur only in subjects homozygous for the variant [20,43]. However, there were no CYP1A1*2A homozygous carriers in our study population, and therefore we cannot rule out a significant role of this variant allele in the development of the disease in our area until larger studies including homozygous individuals are conducted.

The main limitation of the study was the sample size, as it made some subgroups too small to conduct any statistical analysis. It also led to high upper limits of the 95% confidence intervals in the OR calculations. On the other hand, it is of note that the hospital in which the present study was carried out is the only cancer referral centre in this high-incidence region. Therefore, that allowed us to have access to all lung cancer patients, as there were no more patients from this area than those diagnosed and treated at this facility.

## Conclusions

In summary, the results of this initial study show that 2 polymorphisms occurring at the *CYP1A1 *gene locus (Ile462Val and Thr461Val) increase the risk to lung cancer in our population, and especially to SCLC. Moreover, we have shown for the first time that Thr461Val (CYP1A1*4) may be by itself a lung cancer risk factor in Caucasians. However, there must be other factors besides these polymorphisms, smoking, and the impact of a family history of tumours contributing to maintaining the observed uncommonly high lung cancer incidence. A plausible hypothesis that would seem to merit additional study might be the influence of the massive use of pesticides due to intensive agriculture, which is the main economic activity in this area. Other factors, such as dietary habits in the region and their interaction with other genetic polymorphisms have recently been identified as well [44]. Further studies are necessary to fully ascertain the role of gene polymorphisms in high-incidence areas such as that described in the present work.

## Competing interests

The authors declare that they have no competing interests.

## Authors' contributions

CSJ and MJ carried out the molecular genetic studies; AC conceived of the study, participated in its design and performed the statistical analysis. JB and JAC participated in the design of the study and helped with discussion of clinical implications of genetic data. GG coordinated the study, participated in its design and drafted the manuscript. All authors read and approved the final manuscript.

## Pre-publication history

The pre-publication history for this paper can be accessed here:

http://www.biomedcentral.com/1471-2407/10/463/prepub
